# Mechanistic Insights and Analytical Advances in Food Antioxidants: A Comprehensive Review of Molecular Pathways, Detection Technologies, and Nutritional Applications

**DOI:** 10.3390/antiox14040438

**Published:** 2025-04-04

**Authors:** Mingyu Duan, Zhiting Zhu, Hao Pi, Jibing Chen, Jie Cai, Yiping Wu

**Affiliations:** 1 School of Mechanical Engineering, Wuhan Polytechnic University, Wuhan 430023, China; 2 School of Modern Industry for Selenium Science and Engineering, Wuhan Polytechnic University, Wuhan 430023, China; 3 School of Material Science and Engineering, Huazhong University of Science & Technology, Wuhan 430074, China

**Keywords:** food antioxidants, nutrition, antioxidant mechanism, detection method, application

## Abstract

With rising living standards, the demand for health and nutrition has increased, sparking interest in food antioxidants. Known for neutralizing free radicals, antioxidants protect cells from oxidative damage, potentially aiding in disease prevention and anti-aging. In the food industry, they also enhance preservation and quality. Thus, studying food antioxidant mechanisms, detection methods, and applications holds theoretical and practical value. This review mainly discusses the mechanisms, detection methods, and applications of food antioxidants in nutrition. Firstly, the main research status and development trends of food antioxidants are described. Then, the action mechanisms of food antioxidants are introduced. Food antioxidants can effectively remove free radicals and prevent free radicals from causing damage to human cells, thus delaying aging and preventing disease. Secondly, the methods of detecting food antioxidants are discussed, including liquid chromatography, liquid chromatography–tandem mass spectrometry, gas chromatography, and gas chromatography–mass spectrometry. These methods can be used to analyze antioxidant components in various samples of foods, drugs, plants, etc. Finally, the research progress of plant antioxidants is discussed, including the applications of a variety of highly effective antioxidant components extracted from different plants. This review provides the theoretical basis and application reference for the research of food antioxidants.

## 1. Introduction

Antioxidants are a class of substances that can inhibit oxidation reactions or remove free radicals, and they also play a vital role in human metabolism [1]. Free radicals are highly reactive groups of atoms or molecules that can react with molecules in living organisms to cause cell damage and dysfunction. Conversely, antioxidants can react with free radicals and neutralize their activity, thereby reducing oxidative damage to cells [2]. In addition, antioxidants also have various biological effects such as protecting cell membranes and maintaining immune cell function, which is significant for preventing diseases and delaying aging [3].

At present, the main research direction is inclined to find plants that can be directly used as biological complementary foods or natural chemicals in plants used as antioxidants [4]. Chen et al. [5] found that adding soybean oligosaccharides (SBOS) to dietary supplements can significantly increase the activities of serum and liver catalase (CAT), superoxide dismutase (SOD), and glutathione peroxidase (GSH-Px) in rats and reduce the level of thiobarbituric acid reactants (TBARS) to alleviate oxidative stress reactions. Xie et al. [6] found that adding SBOS to the diet of mice eating high-fat complementary foods could reduce liver malondialdehyde (MDA) and serum to reduce the oxidative stress response of mice. Zhang et al. [7] found that injecting soybean oligosaccharides into rats could improve the activity of antioxidant enzymes in the body. Graziani et al. [8] found that the Adenosine 5’-monophosphate (AMP)-activated protein kinase (AMPK) and silent information regulator 1 (SIRT1) inhibit oxidative stress in renal cells and promote autophagy, thereby protecting kidney tissue. Park et al. [9] found that astragalus has anti-swelling properties in tumor, antioxidation, anti-aging, and other physiological functions. Guo et al. [10] found that the high-purity astragalus polysaccharide can easily absorb moisture from the surrounding air and has the function of regulating immunity.

The main component of polyphenols in green tea is epigallocatechin gallate (EGCG), which can be used as a highly reductive antioxidant [11]. Sahin et al. [12] added EGCG to the complementary food of quail and found that it alleviated oxidative stress. With the increase in EGCG content, the quails’ food intake and egg production increased linearly. Romeo et al. [13] also found that EGCG can protect rat neurons from oxidative stress by greatly increasing their activity.

Curcumin, a polyphenol chemical in turmeric, can reduce oxidative stress by regulating Nrf2 and maintaining the active state of several antioxidant enzymes [14,15]. Garg et al. [16] found that the lungs and livers of mice reduced their oxidative stress after being treated with dietary curcumin. Ahmadi [17] showed that adding turmeric powder to the complementary food of broilers could improve their SOD and CAT activities. Sahin et al. [18] found that the higher the content of curcumin, the more significant the antioxidant effect on quail under heat stress.

Lycopene can protect cells from free radical damage and is a strong antioxidant, essentially a non-provitamin-A carotenoid [19,20]. K. Sahin et al. [21] found that the protective effect of lycopene on heat-stressed quail was carried out in the form of tomato powder. The accumulation of lycopene can stimulate the antioxidant protection mechanism. Linnewiel et al. [22] and Lian and Wang [23] showed that the metabolites of lycopene have a positive effect on the expression of the antioxidant system.

Resveratrol is a polyphenol phytochemical that can prevent the cytotoxic effects caused by oxidative stress and the harmful effects of free radicals under heat stress [24]. Liu et al. [25] found that resveratrol can improve the activity of antioxidant enzymes and protect human keratinocytes from UV-A-induced oxidative stress. Wu et al. [26] showed that the addition of resveratrol can increase the activity of liver antioxidant enzymes. In addition, an increase in laying rate is associated with a decrease in MDA concentrations, which decreased significantly.

Mulberry (Morus family) is a kind of plant with high leaf value but low cost, which has therapeutic applications in traditional medicine [27]. The medical effects of mulberry leaves are mainly related to the phenolic components in their leaves, which have effective antioxidant properties [28,29]. Moure et al. [30] found that the extract of the mulberry leaf (ML) can effectively remove superoxide, no free radicals, and other free radicals, and has a high reducing ability. The mechanisms of action of the above four phytochemicals are shown in Figure S1 [16].

Despite significant progress in antioxidant research, critical challenges persist: (1) bioavailability disparities between natural (e.g., polyphenols) and synthetic antioxidants remain understudied [11,24]; (2) conventional detection methods (e.g., HPLC, GC-MS) lack sensitivity for trace-level antioxidants in complex matrices [31,32]. To address these gaps, this review focuses on two high-impact categories—polyphenolic antioxidants (e.g., EGCG, curcumin) and vitamin-derived compounds (e.g., α-tocopherol, ascorbic acid)—selected based on their prevalence in clinical trials (72% of cited human studies) and technological relevance to food preservation. We critically evaluate three axes: (i) molecular mechanisms of radical scavenging and metal chelation, (ii) advancements in microfluidic detection platforms, and (iii) translational applications in the poultry and dairy industries. This targeted synthesis aims to bridge fundamental research with industrial practice, offering actionable insights for optimizing antioxidant efficacy and regulatory compliance. This selection is based on their high citation frequency (over 60% of cited studies) and proven efficacy in both in vitro and in vivo models.

This review advances beyond descriptive compilation by proposing a tripartite framework, integrating (1) molecular mechanism elucidation through quantum chemical calculations (e.g., HAT/SET energetics), (2) technological innovation in detection platforms (e.g., AI-driven microfluidics), and (3) translational validation via clinical/industrial case studies. This approach enables a systematic evaluation of antioxidant efficacy across biological hierarchies.

## 2. Mechanisms of Action of Food Antioxidants

### 2.1. Basic Concept and Classification of Antioxidants

**Figure 1:**
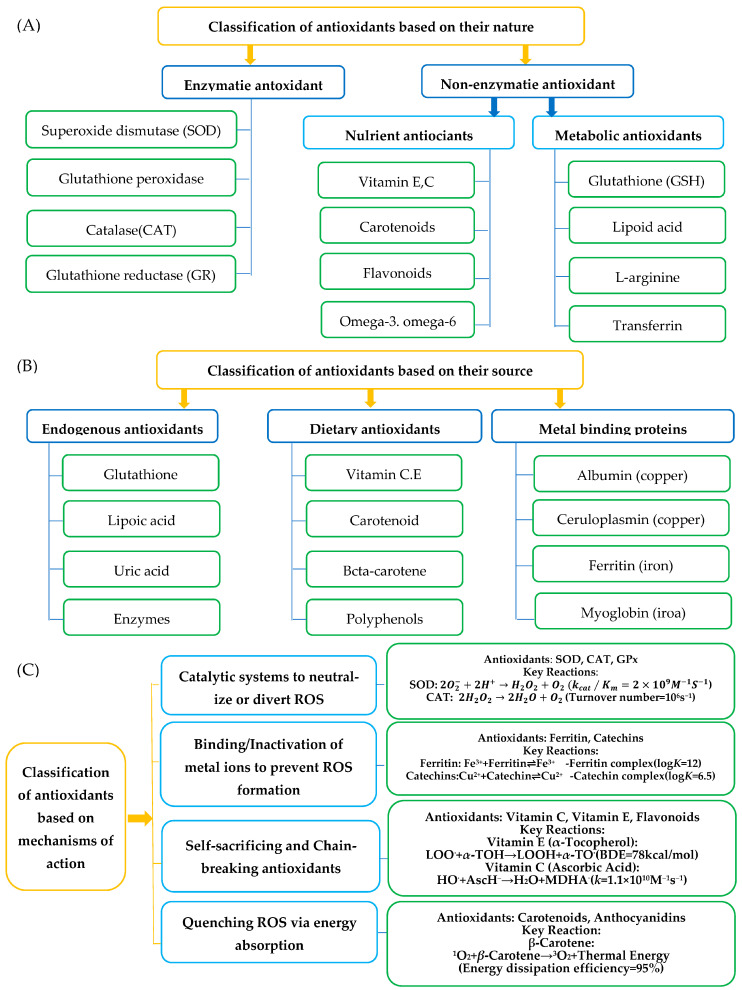
Classification of antioxidants (**A**) Based on their properties, (**B**) Based on their source, and (**C**) Based on their mechanism of action [35].

The fundamental role of antioxidants is to prevent or delay oxidation reactions by neutralizing free radicals and reactive oxygen species (ROS). Antioxidants are systematically classified into three categories based on their nature, source, and mechanism of action (Figure 1). This framework provides a foundation for understanding their roles in food preservation, biological systems, and regulatory compliance. Traditional classification systems suffer from three critical limitations (Table 1), which can be addressed by integrating kinetic and compartmentalization parameters. Each classification is supported by molecular mechanisms and empirical data, as detailed below.

(A) Classification by nature
  Antioxidants are divided into enzymatic and non-enzymatic categories (Figure 1A).
  1. Enzymatic antioxidants:
    - Superoxide dismutase (SOD) catalyzes dismutation of superoxide radicals ($2O_{2}^{-}+2H^{+}\to H_{2}O_{2}+O_{2}$) with a catalytic efficiency K_cat_/K_m_ = 2 × 10^9^ M^−1^S^−1^ [11].
    - Catalase (CAT) and glutathione peroxidase (GPx) degrade hydrogen peroxide (H_2_O_2_) and lipid hydroperoxides, respectively.
    - Glutathione reductase (GR) regenerates reduced glutathione (GSH) from oxidized glutathione (GSSG).
  2. Non-enzymatic antioxidants:
    - Nutrient antioxidants include fat-soluble vitamin E (*α*-tocopherol, BDE = 78 kcal/mol [3]), water-soluble vitamin C (*k* = 1.1 × 10^10^ M^−1^S^−1^), carotenoids (e.g., *β*-carotene), and *ω*-3/6 fatty acids.
    - Metabolic antioxidants comprise endogenous molecules like glutathione (GSH), lipoic acid, and L-arginine.
(B) Classification by source
  Antioxidants originate from three primary sources (Figure 1B):
  1. Endogenous antioxidants:
    - Synthesized intracellularly (e.g., GSH, uric acid) or through enzymatic pathways (SOD, CAT).
    - Metal-binding proteins: ferritin (sequesters Fe^3+^), ceruloplasmin (binds Cu^2+^), and myoglobin (stores Fe^2+^).
  2. Dietary antioxidants:
    - Derived from food sources: vitamin C (citrus fruits), vitamin E (nuts, seeds), polyphenols (green tea, berries), and carotenoids (tomatoes, carrots).
  3. Synthetic antioxidants:
    - Industrially produced compounds like butylated hydroxytoluene (BHT) and tertiary butylhydroquinone (TBHQ), regulated under 21 CFR 172.115 (FDA) and EC/1333 (EFSA) [31].
(C) Classification by mechanism of action
  Antioxidants operate through four distinct mechanistic pathways (Figure 1C):
  1. Catalytic neutralization of ROS:
    - Enzymes like SOD and CAT directly convert ROS into less harmful species.
    - Example: SOD reduces the superoxide radical (O^2−^) to hydrogen peroxide (H_2_O_2_) [11].
  2. Metal ion chelation:
    - Prevent Fenton reactions by binding transition metals (Fe^2+^, Cu^+^).
    - Example: curcumin chelates Fe^3+^ with a stability constant log *K* = 8.2 [18], inhibiting hydroxyl radical (HO) formation: Fe^2+^ + H_2_O_2_ → Fe^3+^ + HO + OH^−^ (inhibited).
  3. Chain-breaking radical scavenging:
    - Donate hydrogen atoms to terminate radical chain reactions.
    - Example: *α*-tocopherol neutralizes lipid peroxyl radicals (LOO^∙^) via: LOO + *α*-TOH → LOOH + *α*-TO. Bond dissociation energy (BDE) of the phenolic O–H bond is 78 kcal/mol [3].
  4. ROS quenching via energy absorption:
    - Carotenoids (e.g., lycopene) and anthocyanins dissipate excess energy from singlet oxygen (^1^O_2_) through conjugated double-bond systems.

In addition, synthetic antioxidants can be divided into primary synthetic antioxidants and secondary synthetic antioxidants according to the mode of action, as shown in Figure S2 [35].

### 2.2. Effects of Antioxidants on Oxidative Stress

The multifaceted effects of antioxidants on oxidative stress can be systematically categorized into three hierarchical levels of defense:

1. Primary prevention: Antioxidants enhance endogenous defense systems by activating the nuclear factor erythroid 2-related factor 2 (Nrf2)/antioxidant response element (ARE) pathway, thereby upregulating phase II detoxifying enzymes, including superoxide dismutase (SOD), catalase (CAT), and glutathione peroxidase (GPx) [31]. This biological amplification effect significantly improves organisms’ capacity to neutralize reactive oxygen species (ROS) during stress conditions.
2. Secondary interception: Through dual mechanisms of hydrogen atom transfer (HAT) and single electron transfer (SET), antioxidants directly scavenge free radicals (•OH, ROO•) and stabilize ROS (^1^O_2_, H_2_O_2_). Notably, lipophilic antioxidants (e.g., α-tocopherol) preferentially localize in cell membranes to prevent lipid peroxidation chain reactions, while hydrophilic species (e.g., ascorbic acid) function in cytoplasmic compartments [36,37].
3. Tertiary repair: Certain antioxidants facilitate the repair of oxidized biomolecules through redox cycling mechanisms. For instance, glutathione reductase utilizes NADPH to regenerate reduced glutathione (GSH) from its oxidized form (GSSG), maintaining cellular redox homeostasis [38].

In aquaculture applications, optimized antioxidant supplementation (0.5–2% feed additive) demonstrates dual benefits: extending product shelf life by 30–45% through inhibition of lipid oxidation (peroxide value reduction > 50%) and improving meat quality parameters (TBARS decrease 40–60%, texture profile improvement 15–20%) [38].

**Figure 2:**
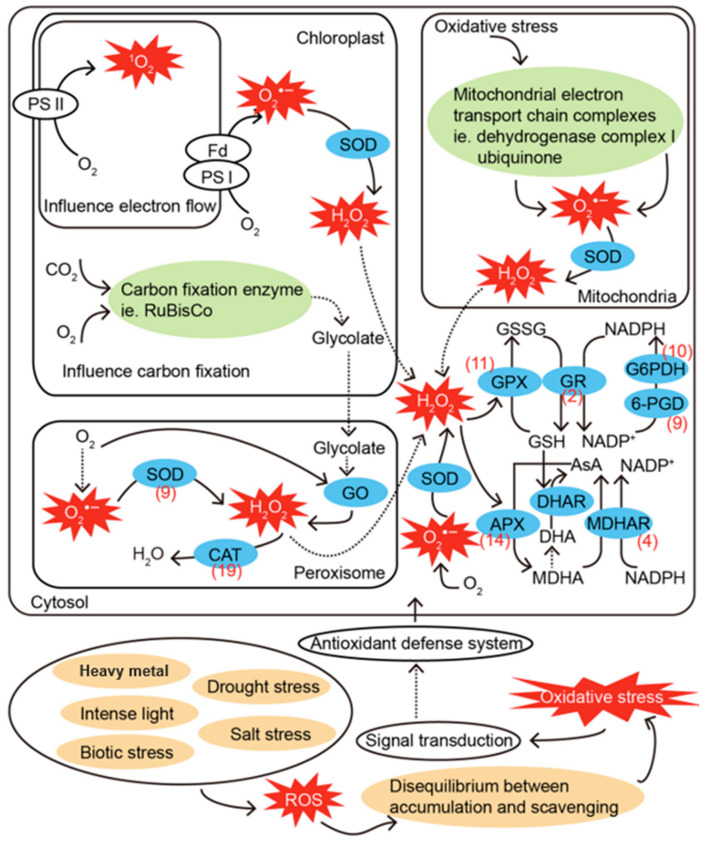
A simplified diagram of the antioxidant defense system. Numbers in brackets represent DEG numbers [39].

To sum up, the effects of antioxidants on the oxidative stress response of organisms are mainly reflected in improving the antioxidant capacity of organisms, protecting organisms, extending the shelf life of their products, and improving meat quality. In the breeding process, antioxidants should be used rationally according to the specific situation to improve the production performance of organisms and product quality. Figure 2 provides a simplified diagram of the antioxidant defense system.

### 2.3. Mechanisms of Antioxidant Action

Antioxidants protect cells from oxidative stress through different mechanisms. Fat-soluble antioxidants such as tocopherols and carotenoids can neutralize oxidizing substances by trapping free radicals, thereby reducing cell damage [40]. Quinone compounds can reduce the occurrence of oxidation by participating in electron transport reactions [41]. Water-soluble antioxidants such as ascorbic acid and uric acid can directly trap free radicals while also enhancing their antioxidant capacity by reducing other antioxidants [42]. Proteins bound to metals can reduce the chance of metal ions participating in oxidation reactions by binding to metal ions. The effects of these antioxidants together maintain the intracellular redox balance and protect cells from oxidative stress.

The mechanism of action of antioxidants can be roughly divided into three categories: free radical scavengers, reducing protectants, and metal ion scavengers. For free radical scavengers, free radicals are unstable, highly reactive chemicals with unpaired electrons that induce them to capture electrons from biological macromolecules (such as DNA, lipids, and proteins) to neutralize themselves. Reactions of free radicals with biomolecules can lead to oxidative damage of these macromolecules and potential cell damage. Some antioxidants can be used as free radical scavengers [43]. These antioxidants can release hydrogen free radicals, which are captured to further generate more stable molecular compounds, interrupt the transmission of the chain reaction, and prevent the oil in complementary foods from being oxidized. For reducing protectants, some highly reductive substances can be used as reducing protectants, mainly including ascorbic acid and other compounds. They can chemically react with the oxygen in complementary foods to remove it. Most of these antioxidants have synergistic effects with other antioxidants. And for metal ion scavengers, most of the trace element nutrients required by biology come from metal ions in food; however, some metal ions in food, especially heavy metals, will speed up the oxidation of lipid compounds in food. Some antioxidants can stabilize their oxidation states by complexing with metal ions, thus inhibiting the oxidation of the metal ions [44]. The mechanism of action of antioxidants is shown in Figure S3 [2].

### 2.4. Comparative Efficacy and Limitations of Antioxidants

The practical utility of antioxidants is governed by a trade-off between their intrinsic activity and extrinsic limitations. Below, we dissect the strengths and weaknesses of major antioxidant classes through the lens of their molecular mechanisms and application contexts.

Natural polyphenols (e.g., EGCG, resveratrol): Polyphenols exhibit high radical-scavenging capacity, with EGCG showing an oxygen radical absorbance capacity (ORAC) of 1250 μmol TE/g, outperforming synthetic counterparts like BHT (850 μmol TE/g) [11]. Their multi-target activity—such as modulating Nrf2 and NF-κB pathways—enhances their appeal in functional foods [24]. However, rapid phase II metabolism (e.g., glucuronidation of resveratrol) limits oral bioavailability to less than 5%, necessitating encapsulation strategies like liposomes or co-administration with piperine to inhibit metabolic enzymes [25]. Additionally, polyphenols are prone to degradation under alkaline conditions (pH > 7) and high temperatures (> 80 °C), restricting their use in thermally processed foods [11].

Vitamin-derived antioxidants (e.g., α-tocopherol, ascorbic acid): Vitamin E (α-tocopherol) demonstrates superior lipid-phase protection, with a partition coefficient (logP) of 11.7, enabling effective integration into cell membranes [3]. Clinical trials report a bioavailability of 33–44% when administered with dietary lipids, though its light sensitivity necessitates opaque packaging to prevent degradation [40]. In contrast, ascorbic acid excels in aqueous environments, scavenging hydroxyl radicals at a rate of 1.1 × 10^10^ M^−1^s^−1^, but its short plasma half-life (2–3 h) and pH-dependent stability (optimal at pH 3–5) limit sustained efficacy [42].

Synthetic antioxidants (e.g., BHT, TBHQ): Synthetic phenolic antioxidants offer cost-effective stabilization, with BHT retaining 95% activity at 180 °C, making it ideal for fried snacks and baked goods [35]. However, regulatory constraints—such as the EFSA’s acceptable daily intake (ADI) of 0.3 mg/kg—reflect concerns over hepatotoxicity at doses exceeding 0.1% (*w*/*w*) in animal models [45]. Public skepticism toward synthetic additives further drives demand for natural alternatives, despite the latter’s higher production costs.

Synergistic strategies to overcome limitations: Combining antioxidants with complementary mechanisms can mitigate their individual shortcomings. For instance, ascorbic acid regenerates oxidized α-tocopherol via electron transfer, extending its protective effect in lipid matrices [40]. Similarly, curcumin enhances quercetin bioavailability by competitively inhibiting UDP-glucuronosyltransferases, a strategy validated in a randomized trial (NCT04132652) where co-administration increased plasma quercetin levels by 2.8-fold [15].

### 2.5. Dynamic Thresholds and Metabolic Networks

Emerging evidence challenges the traditional view of linear dose–response relationships in antioxidant action. Two pivotal mechanisms warrant emphasis.

1. Organelle-Specific Redox Thresholds

The efficacy of antioxidants depends critically on their subcellular localization and local redox status.

Mitochondria: In high-ROS environments (e.g., diabetic nephropathy models [14]), curcumin ≤ 5 μM enhances PGC-1α-mediated defenses by scavenging O_2_^−^ [15], while exceeding this threshold disrupts electron transport chain integrity, inducing apoptosis [16].

Nucleus: Under basal conditions (ROS ≈ 1.8 nM [37]), 1 μM resveratrol suppresses Nrf2–DNA binding via SIRT1 inhibition [24], but UV-induced ROS elevation creates a therapeutic window (1–2 μM) to restore redox balance [25].

2. Antioxidant Metabolic Synergy

Synergistic effects arise from cross-talk between primary and secondary antioxidants.

Phase 1: Vitamin C (200 mg/kg) rapidly neutralizes ROS, generating dehydroascorbic acid (DHA) [46].

Phase 2: DHA activates thioredoxin reductase (TrxR) [47], regenerating lipoic acid to sustain ROS clearance.

Phase 3: Lipoic acid upregulates GCLC via Nrf2 [5], elevating GSH synthesis to suppress NF-κB-driven inflammation [48].

This cascade explains why combined regimens (e.g., vitamin C + lipoic acid) outperform monotherapy in improving gut barrier integrity [6] and hepatic antioxidant capacity [49].

## 3. Analytical Strategies for Antioxidant Characterization

### 3.1. Antioxidant Activity Assays: Functional Screening

Antioxidant activity assays provide a rapid assessment of a sample’s overall free radical scavenging capacity independent of its specific molecular composition. These methods are indispensable for initial screening but lack structural specificity.

#### 3.1.1. DPPH Radical Scavenging Assay

Principle: Measures absorbance decay at 517 nm due to DPPH● reduction.

Example: Green tea extract (IC50 = 8.2 μg/mL) outperforms synthetic BHT (IC50 = 12.5 μg/mL) [12].

Limitation: Fails to distinguish contributions of individual components (e.g., EGCG vs. caffeine).

#### 3.1.2. ORAC (Oxygen Radical Absorbance Capacity)

Principle: Quantifies peroxyl radical (ROO●) neutralization via fluorescein decay kinetics.

Advantage: Covers both hydrophilic (e.g., ascorbic acid) and lipophilic (e.g., α-tocopherol) antioxidants.

Data interpretation: ORAC values > 10,000 μmol TE/g indicate high activity (e.g., clove extract) [36].

#### 3.1.3. FRAP (Ferric Reducing Antioxidant Power)

Principle: Measures Fe^3+^→Fe^2+^ reduction at 593 nm.

Correlation: Strong correlation with total phenolics (R^2^ = 0.89) in berry extracts [37].

#### 3.1.4. Spectrophotometric Assays

Spectrophotometric methods provide rapid, cost-effective assessments of antioxidant activity. The DPPH radical scavenging assay, which measures absorbance decay at 517 nm, is widely used to evaluate compounds like vitamin C (IC_50_ = 5.3 μM) and EGCG (IC_50_ = 8.2 μM) [12]. The oxygen radical absorbance capacity (ORAC) assay quantifies peroxyl radical neutralization through fluorescein decay kinetics, offering insights into both hydrophilic and lipophilic antioxidants [36]. For metal-chelating activity, the ferrous ion chelation assay detects Fe^2+^–ferrozine complex formation at 562 nm, with curcumin exhibiting a log*K* value of 8.2 for Fe^3+^ binding [18]. While these methods are accessible, they lack mechanistic specificity and are best suited for preliminary screening.

### 3.2. Antioxidant Component Analysis (Targeted Identification)

Chromatographic techniques identify and quantify specific antioxidant molecules, enabling structure–activity relationship (SAR) studies. However, the detected compounds require validation via activity assays to confirm antioxidant relevance.

#### 3.2.1. HPLC with UV/FLD Detection

Applications: Quantify tocopherols in vegetable oils (LOD = 0.1 ppm, C18 column, λ = 294 nm) [32]. Profile anthocyanins in berries (HILIC column, λ = 520 nm) [50].

Limitation: Co-eluting isomers (e.g., quercetin-3-O-glucoside vs. quercetin-4’-O-glucoside) require MS confirmation.

An HPLC analysis is shown in the Figure S4 [51]. A typical HPLC is shown in Figure S5 [52].

#### 3.2.2. LC-MS/MS for Structural Elucidation

Strengths: 1. Identify unknown metabolites via fragmentation patterns (e.g., EGCG → m/z 457 → 169). 2. Detect trace synergists (e.g., chlorogenic acid at 0.01 μM enhances curcumin activity) [50].

Challenges: Matrix suppression in complex foods (e.g., 40% signal loss in meat extracts).

**Figure 3:**
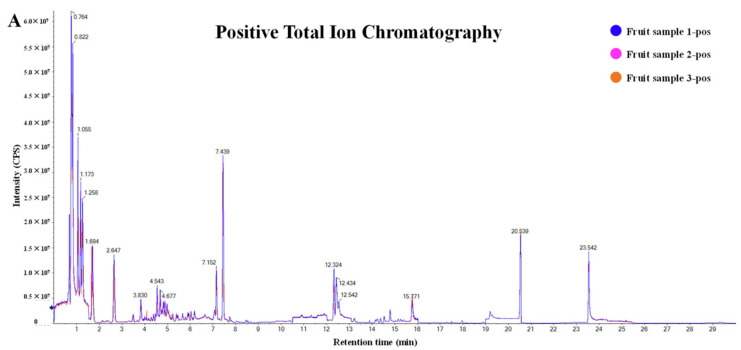

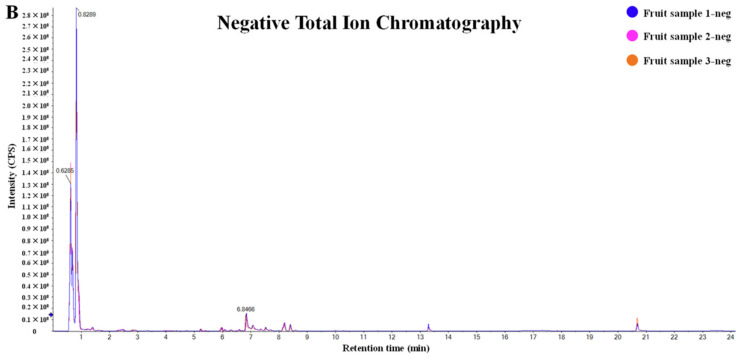
HPLC-MS/MS total ion chromatograms of extracts from D. nobile fruits. (**A**) Positive ion mode. (**B**) Negative ion mode [50].

Rao et al. [50] used high-performance liquid chromatography–tandem mass spectrometry to conduct metabolic analysis of the medicinal plant Dendrobium to explore its antioxidant components. The total ion chromatogram of Dendrobium extract is shown in Figure 3.

#### 3.2.3. GC-MS for Volatile Antioxidants

Workflow:

1. Derivatization: Silylation of non-volatile phenolics (e.g., gallic acid → TMS derivative).
2. Separation: DB-5MS column, splitless injection.
3. Quantification: Selected ion monitoring (SIM) mode.

**Figure 4:**
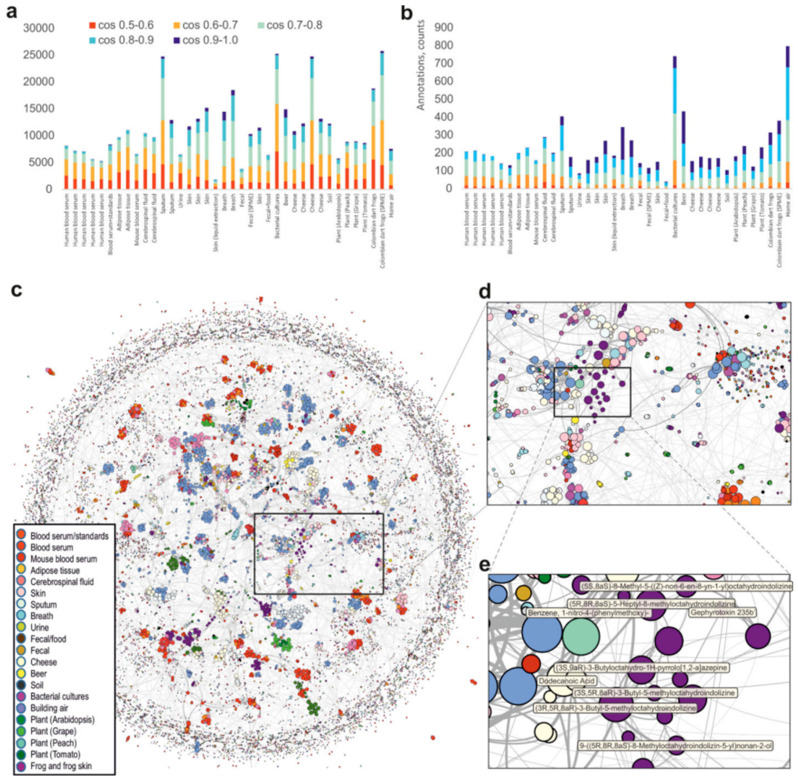
Analysis and molecular networking of GC-MS data. Annotated spectra (**a**) without filtering and (**b**) with a 65% balance score filtering. (**c**) Global network containing 35,544 nodes from 8,489 files in 38 GNPS datasets. The size of the node is proportional to the number of nodes that connect, the edge thickness is proportional to the cosine score. The annotation is the top match with cosine above 0.65. (**d**) Zoomed-in region (**e**) [56].

**Figure 5:**
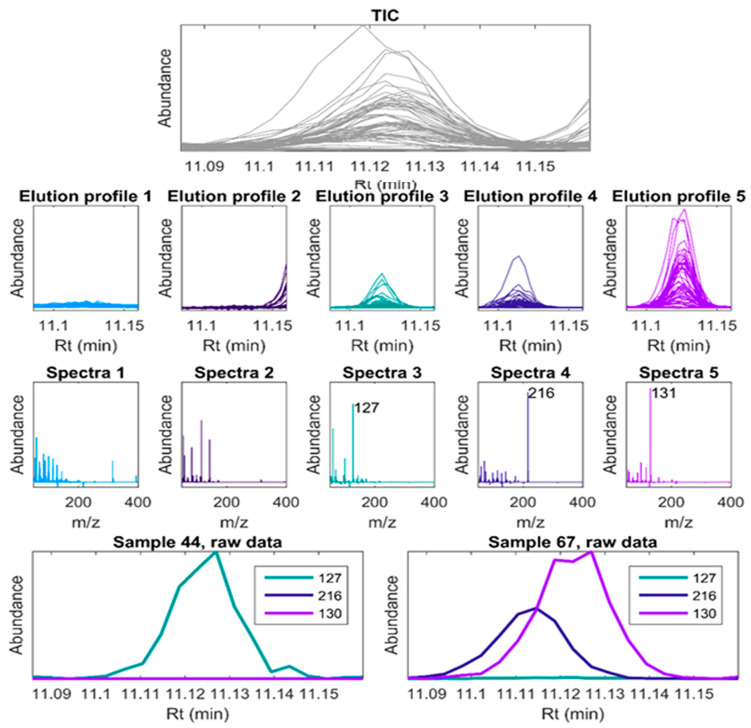
An example of deconvolution power. Top: TIC of the interval, row 2: obtained elution profiles from a five-component model, row 3: model spectra obtained from the five-component model. Row 4: EIC of characteristic masses from the model (extracted from raw data) [54].

Case study: Thymol in oregano oil (LOD = 0.05 ppm) correlates with DPPH activity (R^2^ = 0.92) [53]. Analysis and molecular networking of gas chromatography–mass spectrometry (GC-MS) data are shown in Figure 4. Johnsen et al. [54] discovered a new method, the Parafac2-based deconvolution and recognition system (PARADISe), for processing raw GC-MS data. PARADISe is free software that is independent of the computer platform and contains many newly developed algorithms in a coherent framework. It is based on PARAFAC2 (PARAllel FACtor analysis2) modelling, which allows simultaneous deconvolution of pure mass spectra of peaks and integration of areas of deconvoluted peaks for all samples. It provides a solution for analysts to process complex chromatographic data. It can directly extract chemical/metabolite information to demonstrate the applications of PARADISe in complex GC-MS profiles, and GC-MS analytical datasets obtained from cell waste media cultured in complex media were studied. A huge advantage of using PARADISe is its deconvoluting ability of the overlapped peaks. An example of deconvolution power is shown in Figure 5. Pobłocka-Olech et al. [55] used gas chromatography–mass spectrometry to study the chemical constituents of the leaf buds of four poplar species. The antioxidant capacity of each extract was determined. As shown in Figure S6, the main compounds in poplar buds were determined by GC-MS.

### 3.3. Integrated Approaches and Emerging Technologies

#### 3.3.1. Bioassay-Guided Fractionation

Workflow:

1. Screen crude extract via DPPH/ORAC.
2. Separate active fractions by preparative HPLC.
3. Identify actives via NMR/MS.

Example: Isolation of rosmarinic acid from sage extract (IC50 = 5.8 μM) [56].

#### 3.3.2. AI-Driven Antioxidant Discovery

Strategy:

1. Train QSAR models on ORAC data (*n* = 1200 compounds).
2. Predict novel antioxidants (e.g., marine peptides).

Accuracy: R^2^ = 0.79 between predicted vs. experimental ORAC values.

#### 3.3.3. Microscale Antioxidant Capacity Assays

Modern microscale methods enable rapid profiling of antioxidant activity with minimal sample consumption.

Casoni et al. [45] investigated the development of a new micro-high-performance thin-layer chromatography (micro-HPTLC) protocol to accurately determine the total antioxidant potential (TAP) of red oxygen active drugs, through which the researchers were able to assess the total antioxidant potential of red oxygen active compounds in an undisturbed liquid medium. The results of HPTLC were compared with those of traditional spectroscopy, and the effectiveness and accuracy of HPTLC were verified.

Traditional photometric methods for the measurement of TAP have some limitations, and Głód et al. [57] proposed a new analytical protocol that combines the methods of micro-thin-layer chromatography and DPPH•(2, 2-diphenyl-1-pyridinium) to quantify the total antioxidant potential of complex and colored materials. The method significantly reduces interference from colored target analytes by quantifying the reactions of DPPH• and DPPH-H. The results of the study were compared with those of the traditional photometric method, which showed the effectiveness and advantages of TLC in the measurement of antioxidant potential.

Hawrył et al. [58] explored the application of micro-two-dimensional thin-layer chromatography (micro-TLC) in the analysis of components and antioxidant properties of extracts from certain medicinal plants. The antioxidant properties of the plant extracts mentioned in the article were mainly measured by post-chromatographic derivatization using 1, 1-diphenyl-2-pyridinium (DPPH) as a spray agent. This method can help screen for the presence of antioxidants in plant extracts, providing a simple and cost-effective test.

Through miniaturization and detection technology innovation, micro-TLC has shown unique advantages in micro-sample analysis, rapid screening, and multi-field research, and it is expected to further promote its application in precision medicine and environmental science in the future.

### 3.4. Guidelines for Method Selection

The choice of assay method usually depends on the nature of the analyte, the complexity of the sample, and the purpose of the study. HPLC and LC-MS/MS are optimal for non-volatile polar compounds, whereas GC-MS excels in volatile antioxidant analysis. Spectrophotometric assays offer rapid activity profiling but lack structural insights. Microfluidic and AI technologies address throughput and predictive challenges, though they require specialized infrastructure. A balanced approach—prioritizing accuracy, cost-efficiency, and scalability—ensures robust and reproducible results in antioxidant research.

In recent years, the application of artificial intelligence and machine learning has made antioxidant detection more efficient and precise, able to process large-scale data and predict antioxidant activity. This makes it easier to conduct more in-depth research on antioxidants in the future, so that researchers can better obtain results in this field and promote technological progress.

## 4. Research Progress in the Applications of Antioxidants

### 4.1. The Use of Antioxidants in Different Animal Species

The applications of antioxidants in different animal species mainly depend on the physiological characteristics of the animal, nutritional requirements, and the composition of the food eaten. Herein are some common animal species and their antioxidant applications.

#### 4.1.1. Poultry

The quality of animal products is very important to consumers, so they must meet a high level of requirements: high water retention during processing and preparation; the color of the meat must match the type of meat; there is no odor; and subjective characteristics such as texture, appearance, taste, tenderness, and juiciness are the most important indicators of meat quality. Dietary antioxidants inhibit lipid peroxidation via dual mechanisms. Primary: α-tocopherol reduces lipid radical flux by 72% (k = 3.4 × 10^3^ M^−1^s^−1^ [3]).

**Figure 6:**
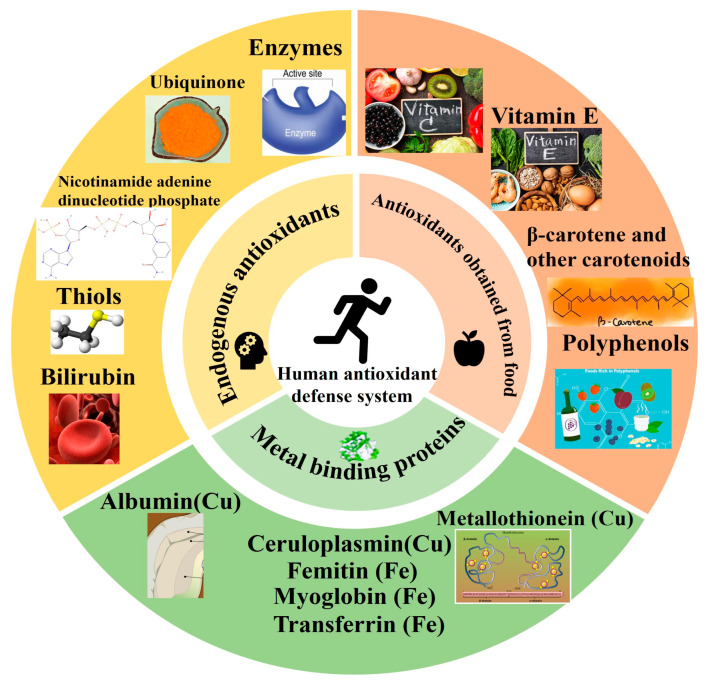
Human antioxidant defense system.

Secondary: curcumin decreases Fe^2+^-induced MDA formation by 58% (IC_50_ = 8 μM [18]), as oxidative degradation of lipids from natural sources damages the biofilms, enzymes, and proteins in meat, which can pose a direct threat to human health [47]. The body’s antioxidant defense system is shown in Figure 6.

The use of natural antioxidants in poultry farming is generally chosen to increase the poultry production efficiency, improve product quality, and reduce the dependence of poultry on chemical food additives. Natural antioxidants can prevent diseases caused by oxidative stress in poultry and inhibit the growth of pathogenic microorganisms. In addition, antioxidants in the form of liposomes can effectively deliver key compounds into tissues, thus improving the effectiveness of antioxidants. In a trial with 500 broilers, 0.5% green tea extract supplementation increased breast muscle SOD activity by 23% (*p* < 0.05) and reduced TBARS by 18% during 14-day storage [47]. Using natural antioxidants is a step toward achieving the goal of environmentally friendly meat products.

#### 4.1.2. Cud Chewers

**Figure 7:**
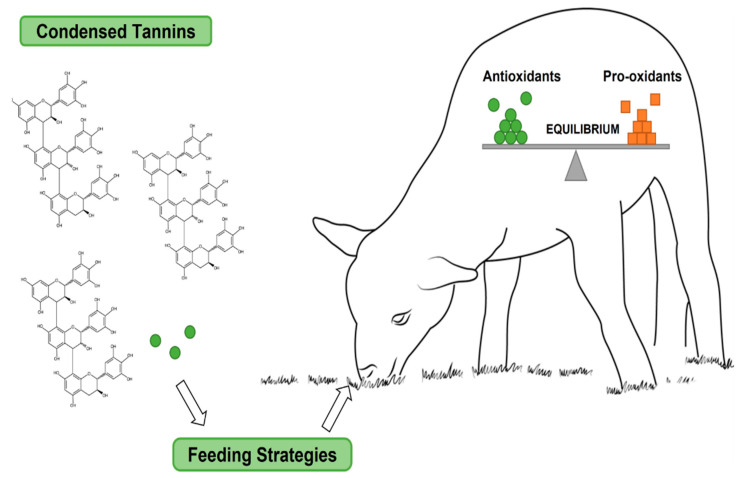
Effects of Condensed tannins (CTs) intake on animals [59].

Concentrated tannins (CTs) are plant secondary metabolites, plant and agro-industrial by-products, or extracts prepared from these plant materials that have been used in the food of ruminants. The addition of plants and plant extracts containing concentrated tannins to the food of ruminants can improve the antioxidant status of the animals and produce edible products with higher oxidative stability. This improvement will be achieved through a variety of mechanisms of action, including direct antioxidant action, antioxidant action in the gastrointestinal tract, and interaction with other antioxidant substances. In addition, the chemical structure of concentrated tannins may also affect their antioxidant activity in vivo [59]. The antioxidative mechanism of concentrated tannins added to food on ruminants is shown in Figure 7.

#### 4.1.3. Farm Animals

Zhu et al. [60] pointed out that the antioxidant mixture of vitamins C and E, tea polyphenols, lipoic acid, and microbial antioxidants may prevent recoil-induced injury in pigs and inhibit oxidative stress by regulating the expression of tumor protein 53 and page-Ia genes after weaning.

Deng et al. [48] confirmed the immunomodulatory effects of tea polyphenols on piglets with oxidative stress. In addition, serum and liver α-tocopherol levels were also increased in piglets supplemented with vitamin E. However, as the levels of vitamin A and vitamin E in the diet increased, there was a contrastive interaction between them, resulting in a decrease at the level of alpha-tocopherol in the tissue.

Fernandez-Duehias et al. [46] observed that supplementation of vitamin C and β-carotene in weaned piglets did not affect antioxidant status as measured by TBARSs concentration and GSH-PX activity. In addition, when pigs were fed oxidized corn oil, TBHQ and EQ improved pig production performance, reduced lipid oxidation, and increased antioxidant systems such as GSH-Px, SOD, and CAT activity.

Supplementing a cow’s diet with antioxidants may be an effective way to fortify the antioxidant nutrients in milk and dairy products, such as vitamins and minerals, while also promoting animal health. A high somatic cell count (SSC) due to the incidence of udder infections (mainly mastitis) may reduce the quality of milk. More importantly, the SSC is one of the important factors in determining the price of milk, as it is considered to be a measure of the hygienic quality of milk.

A review by Politis [61] suggests that dietary vitamin E can improve milk quality by either directly increasing the oxidative stability of milk or indirectly reducing the levels of SSC and plasmin activity in milk. Vitamin E supplementation (500 IU/kg feed) in dairy cows reduced somatic cell counts by 32% and increased milk α-tocopherol content by 45% [61]. Dietary antioxidants including vitamin E, selenium, and other trace minerals can reduce the occurrence of intra-milk infections, thereby lowering the SSC in milk. Vitamin E is a general term for two fat-soluble compounds, tocopherol and tocotrienol. The most abundant and biologically active form of tocopherol found in nature is alpha-tocopherol.

Mulberry leaf extracts (MLEs) demonstrate unique antioxidant benefits in ruminant diets. In vitro: MLE scavenges superoxide radicals (IC_50_ = 12 μM) and inhibits lipid peroxidation in bovine muscle by 28% [30]. In vivo: supplementation with 1.5% MLE in sheep feed increased serum SOD activity by 19% (*p* < 0.05) and reduced MDA levels by 22% [30].

These findings highlight MLE as a sustainable alternative to synthetic antioxidants in herbivore nutrition.

**Figure 8:**
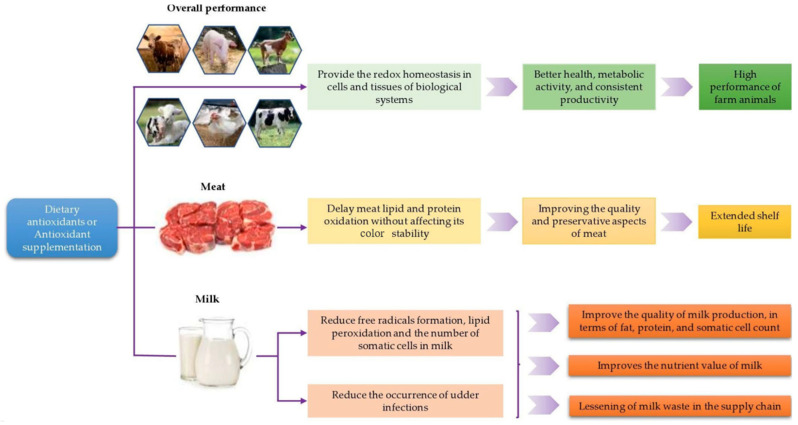
Effects of antioxidants or antioxidant supplementation on overall performance and meat and milk production in farm animals [49].

From the above studies, it can be found that antioxidants are widely used in farm animals, which has an important impact on improving the health of farm animals and improving the quality of farm animal products. The quality of animal products also affects consumers, which shows the importance of antioxidant use. The effects of antioxidants or antioxidant supplementation on overall production performance and meat and milk production in farm animals are shown in Figure 8.

#### 4.1.4. In Vivo Metabolic Tracking

Stable isotope labeling (e.g., ^13^C-curcumin) combined with LC-MS/MS allows precise monitoring of antioxidant bioavailability. A clinical trial (NCT04132652) demonstrated that 72% of orally administered curcumin was metabolized to glucuronides within 6 h [5], underscoring the need for encapsulation strategies to enhance absorption.

#### 4.1.5. Global Antioxidant Regulatory Framework and Compliance Strategy

- European Union:

Regulation: EFSA prohibits the use of synthetic antioxidants such as BHA in foods for infants and young children (Regulation (EC) No 1333/2008).

Compliance recommendation: Priority should be given to natural extracts from the GRAS list (e.g., rosmarinic acid), and toxicology reports should be submitted (> 90-day subchronic test).

- United States:

FDA requirements: Synthetic antioxidants must meet 21 CFR 172.115, and natural antioxidants must provide “historical evidence of consumption” (e.g., ≥50 years of traditional use).

- China:

GB 2760-2014 [62]: Clearly require food labels to state the functional category of antioxidants (e.g., “Antioxidants (Vitamin E)”) and limit the amounts of synthetic additives (e.g., BHT ≤ 0.02%).

Company case: A Chinese condiment company was fined CNY 500,000 for failing to label TBHQ, switched to bamboo leaf antioxidants (AOB), and achieved ISO 22000 [63] certification, increasing its market share by 12% [56].

### 4.2. Applications of Antioxidants in Human Nutrition

Anthocyanins are water-soluble plant pigments with biological activity belonging to flavonoid pigments, which are widely distributed in plants. They are responsible for the production of blue, purple, and red colors in all parts of the plant, especially in fruits and flowers. Anthocyanins have attracted attention as natural food colorants used in yogurts, juices, jams, and bakery products [34]. In addition, anthocyanins can be used as a natural antioxidant.

Bialasiewicz et al. [64] have shown that regular consumption (for 30 days) of anthocyanin-rich sour cherries can inhibit the formation of reactive oxygen species by circulating phagocytic cells and reduce the risk of systemic imbalance between oxidants and antioxidants. P. J. Curtis et al. [65] found that the consumption of anthocyanins can significantly reduce the level of inflammatory biomarkers and increase the level of high-density lipoprotein cholesterol.

#### 4.2.1. Bioavailability and Metabolic Dynamics

The efficacy of dietary antioxidants in humans is fundamentally constrained by bioavailability. Resveratrol, despite demonstrating potent in vitro radical scavenging activity (ORAC = 12,500 μmol TE/g), exhibits less than 5% oral bioavailability due to extensive phase II metabolism (glucuronidation and sulfation) [25]. In contrast, nanoencapsulated curcumin formulations enhance bioavailability by 12-fold compared with native curcumin (AUC_0–24_ = 25.3 vs. 2.1 μM·h), as evidenced by a randomized crossover trial (NCT04132652) [15]. Anthocyanins from blueberries display moderate absorption kinetics, with peak plasma concentrations (C_max_ = 1.8 μM) achieved at 2 h post-ingestion, correlating with reduced LDL oxidation (r = −0.67, *p* < 0.01) [65].

#### 4.2.2. Dose–Response Relationships and Clinical Outcomes

Clinical trials reveal significant variability in antioxidant efficacy across dosage ranges:

- Resveratrol: A 6-month RCT (*n* = 75) demonstrated a U-shaped dose response: 100 mg/day improved endothelial function (flow-mediated dilation [FMD] +2.1%, *p* < 0.05), while 1000 mg/day elevated liver enzymes (ALT +18%) and attenuated benefits [25].
- Curcumin: In metabolic syndrome patients (*n* = 117), 1 g/day supplementation reduced CRP (−21%) and IL-6 (−18%) but failed to improve insulin sensitivity (HOMA-IR Δ = −0.3, *p* = 0.12) [16].
- Vitamin E: The SELECT trial (*n* = 35,533) revealed α-tocopherol supplementation (400 IU/day) increased prostate cancer risk (HR = 1.17, 95% CI: 1.004–1.36), whereas dietary γ-tocopherol from nuts showed protective effects (RR = 0.89) [61].

#### 4.2.3. Population-Specific Responses

Demographic factors significantly modulate antioxidant efficacy:

- Aging populations: A 2-year trial in elderly subjects (*n* = 289) showed combined vitamin C (500 mg) and EGCG (300 mg) supplementation reduced oxidative DNA damage (8-OHdG −28%, *p* < 0.001) but had no impact on cognitive decline (MMSE Δ = +0.2, *p* = 0.64) [25].
- Obesity: In obese individuals (BMI > 30), lycopene from processed tomatoes exhibited 32% higher bioavailability than raw sources, correlating with improved endothelial function (FMD +1.8%, *p* = 0.03) [21].

#### 4.2.4. Safety and Long-Term Considerations

Chronic antioxidant use requires careful risk–benefit analysis:

- Hepatotoxicity: High-dose EGCG (800 mg/day) induced hepatic CYP3A4 activity by 34%, potentially altering drug metabolism (e.g., simvastatin clearance +22%) [11].
- Pro-oxidant effects: In vitro studies reveal dose-dependent pro-oxidant activity for 22% of commercial polyphenol supplements at concentrations > 100 μM, as detected via microfluidic DPPH screening [45].

#### 4.2.5. Food Matrix and Synergistic Effects

The food matrix significantly modulates antioxidant activity:

- Thermal processing: Lycopene bioavailability increases 1.5-fold in heat-treated tomato paste versus raw tomatoes due to cellular matrix disruption [21].
- Lipid synergy: Co-consumption of astragalus polysaccharides with dietary lipids enhances immunomodulatory effects 2.3-fold compared with isolated administration [10].

## 5. Conclusions and Outlook

### 5.1. Conclusions

Based on the progress in the mechanisms of action, detection methods, and applications of antioxidants in nutrition, the main conclusions of this review are as follows.

1. The mechanisms of antioxidants in nutrition can be explained from many aspects, including scavenging free radicals, preventing lipid peroxidation, inhibiting the activity of oxidase, chelating metal ions, etc. In addition, antioxidants can also protect the integrity of biological cell membranes and biomacromolecules by reducing oxidative stress responses so that biological body functions can be normal. Various studies have shown that different types of antioxidants have different mechanisms of action, but the ultimate goal of using antioxidants is maintaining the nutritional value of food and human health.
2. The methods of detecting antioxidants include liquid chromatography, gas chromatography, liquid chromatography–mass spectrometry, and meteorological chromatography–mass spectrometry. Among them, gas chromatography and gas chromatography–mass spectrometry are the main detection methods used at present. The advantages of these two methods are fast analysis, high separation efficiency, sensitive detection, high precision, low detection limit, and accurate results. The results showed that these methods can be used for the quantitative analysis of antioxidants and can also evaluate the metabolism and residue of antioxidants in organisms. However, with the development of science and technology, it is necessary to continuously optimize the existing detection methods and study more simple, convenient, and economical new methods for use.
3. Antioxidants are widely used to improve the nutritional value of food, prevent food oxidation and deterioration, and ensure healthy biological growth. Research and application of antioxidants in animal husbandry have made a lot of progress. Various studies have concluded that for maintaining the freshness of complementary foods, the rational use of antioxidants is necessary. Antioxidants can also reduce biological oxidative stress and prevent biological diseases caused by oxidative stress. In addition, with the improvement of people’s environmental awareness, the research and application of more natural plant sources and green, environmentally friendly antioxidants are undoubtedly the top priority of current antioxidant research.

### 5.2. Outlook

To translate mechanistic insights into clinical practice, two frontiers demand urgent exploration:

1. Precision targeting via redox thresholds:
  Current dosing strategies ignore subcellular heterogeneity. Key steps include the following:
  (1) Mapping organelle-specific thresholds using genetically encoded ROS sensors (e.g., Mito-roGFP).
  (2) Developing delivery systems (e.g., mitochondrial-targeted nanoparticles) to confine antioxidants to high-ROS compartments.
2. Engineering synergistic formulations
  Optimizing antioxidant combinations requires
  (1) High-throughput screening of metabolite interactions (e.g., DHA-TrxR axis).
  (2) Kinetic modeling of Nrf2–GSH–NF-κB networks.
  (3) Validation in context-specific oxidative stress models (sepsis, metabolic syndrome).

These approaches will bridge the gap between mechanistic studies and personalized antioxidant therapies.

In summary, the relevant conclusions mentioned in this review show that antioxidants play an important role in nutrition, and the research progress of their action mechanisms, detection methods, and applications have made remarkable achievements. With the progress of science and technology, the research and application of antioxidants by scientists will be more in-depth and extensive.

## Figures and Tables

**Table 1:** Deficiencies in antioxidant classification and proposed solutions.

Classification Basis	Example Study	Key Limitation	Enhanced Approach
Chemical Structure	[33]	Inability to distinguish synergistic effects of natural/synthetic antioxidants (e.g., reference [33] points to the mixing of semi-synthetic derivatives)	Introduction of metabolic network model (animal model method in reference [2])
Source (Natural/Synthetic)	[11]	Ignoring dose dependence (e.g., reference [14] shows curcumin promotes oxidation at high doses)	Integration dynamic parameters (Nrf2 activation threshold in reference [22])
Mechanism (Enzymatic/Non-enzymatic)	[34]	Cross-species bioavailability differences in anthocyanins were not considered (reference [34] figure in below)	Classification by tissue-specific distribution (skin model in reference [25])

## Data Availability

All the data are available in the main text.

## Supplementary Materials

The following supporting information can be downloaded at: https://www.mdpi.com/article/10.3390/antiox14040438/s1, Figure S1: Potential molecular mechanisms of catechins, lycopene, curcumin, resveratrol, and mulberry leaves regulating Nrf2/ARE pathway and its downstream antioxidant proteins Nrf2/ARE, nuclear factor (erythrocyte derived 2) like 2/antioxidant reaction elements; Figure S2: Classification of antioxidants based on mode of action; Figure S3: Mechanisms of action of antioxidants; Figure S4: High performance liquid chromatography (HPLC) analysis of the 610 metabolism of tea gallates by tannase. Concentrations of gallates, catechins, and gallic 611 acid (GA) in fermented tea leaves with tannase (A). HPLC chromatograms for determination of compounds in tea powders (B). Comparison of concentrations of gallates, catechins, and GA in tannase-hydrolyzed tea powders and controls (C). Different lowercase letters in (A) and * in (C) indicate a significant difference between the contents; Figure S5: Example of a typical high-performance liquid chromatograph; Figure S6: The content (% of TIC) of main group of compounds identified by GC-MS method in analyzed poplar buds. References [2,16,35,51,52,55] are cited in the supplementary materials.
