# Supplementary material for: Mechanistic Insights and Analytical Advances in Food Antioxidants: A Comprehensive Review of Molecular Pathways, Detection Technologies, and Nutritional Applications
Source: Antioxidants (Basel). 2025 Apr 4;14(4):438. doi: 10.3390/antiox14040438 (PMC12024333; doi:10.3390/antiox14040438)
Supplement: Supplementary file 1 [file antioxidants-14-00438-s001.zip › antioxidants-3475948-supplementary.pdf]

Supplementary Information

Research progress on the mechanisms, detection methods, and applications of food antioxidants to nutrition

Mingyu Duan [1], Zhiting Zhu [1], Hao Pi [1], Jibing Chen [1,*], Jie Cai [2], and Yiping Wu [3]

[1] School of Mechanical Engineering, Wuhan Polytechnic University, Wuhan 430023, China; myduan02722@163.com (M.D.); zhitingzhu0331@163.com (Z.Z.); pihao001@163.com (H.P.)
[2] School of Modern Industry for Selenium Science and Engineering, Wuhan Polytechnic University, Wuhan 430023, China; caijievip@whpu.edu.cn (J.C.)
[3] School of Material Science and Engineering, Huazhong University of Science & Technology, Wuhan 430074, China; ypwu@mail.hust.edu.cn
* Correspondence: jbchen@whpu.edu.cn(J.C)

* Corresponding author: Jibing Chen
School of Mechanical Engineering
Wuhan Polytechnic University
Wuhan 420023, Hubei, China
E-mail address: jbchen@whpu.edu.cn

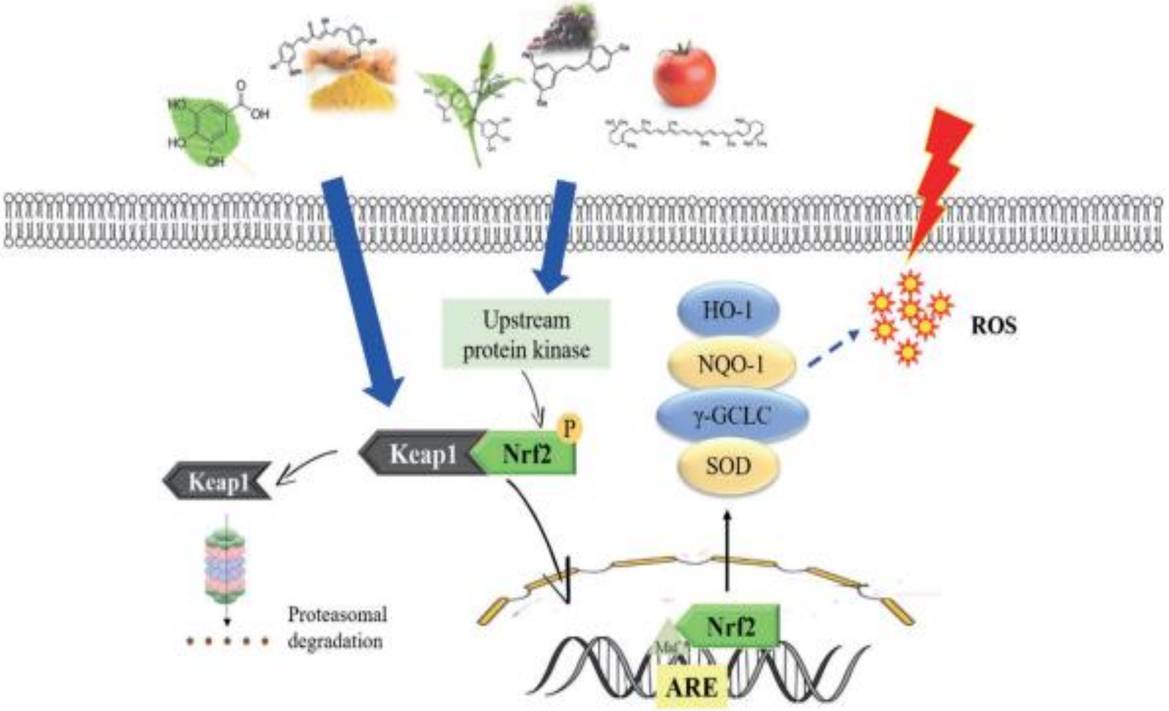

Figure S1. Potential molecular mechanisms of catechins, lycopene, curcumin, resveratrol, and mulberry leaves regulating the Nrf2/ARE pathway and its downstream antioxidant proteins Nrf2/ARE, nuclear factor (erythrocyte derived 2)-like 2/antioxidant reaction elements (Garg & Maru, 2009).

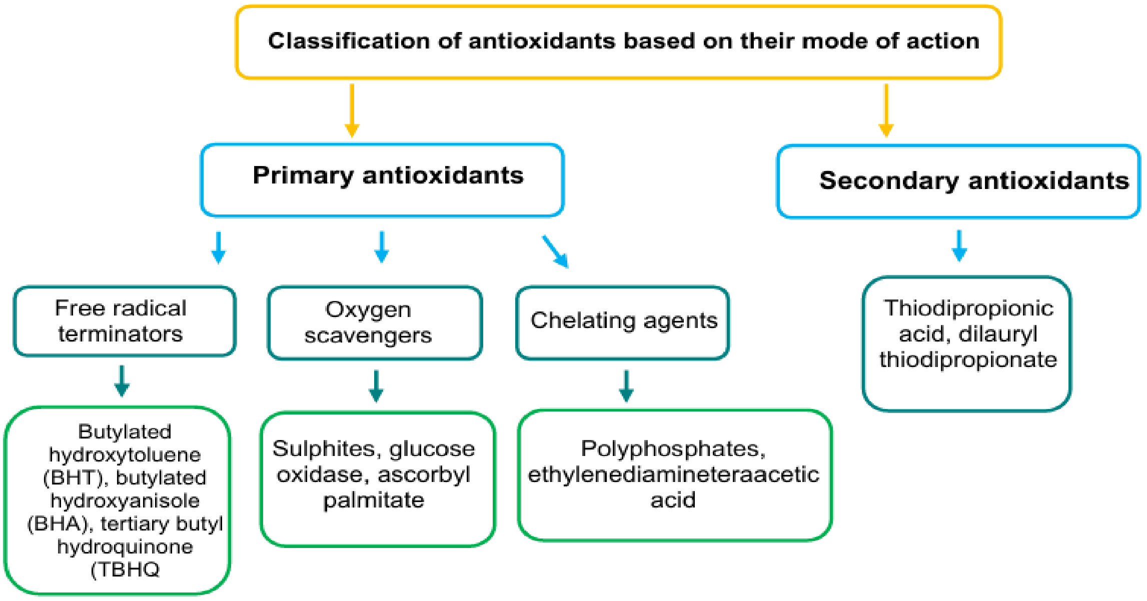

Fig. S2. Classification of antioxidants based on mode of action (Jamshidi-Kia, Wibowo, Elachouri, Masumi, Salehifard-Jouneghani, Abolhassanzadeh, & Lorigooini. 2020).

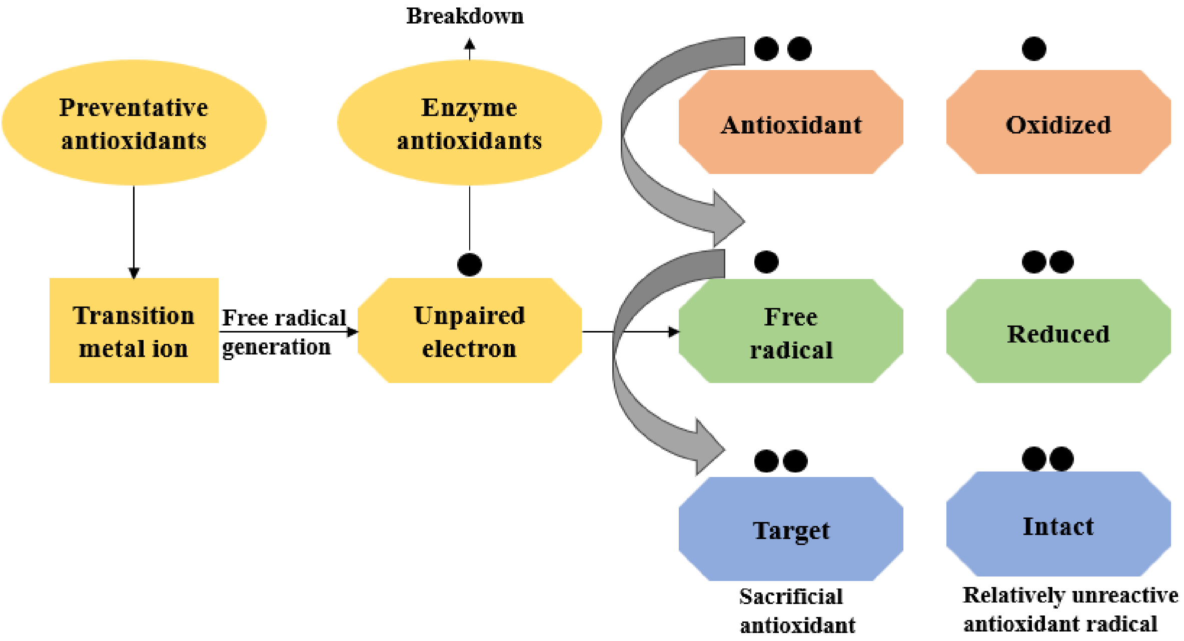

Figure S3. Mechanisms of action of antioxidants (Salami, Guinguina, Agboola, Omede, Agbonlahor, & Tayyab, 2016).

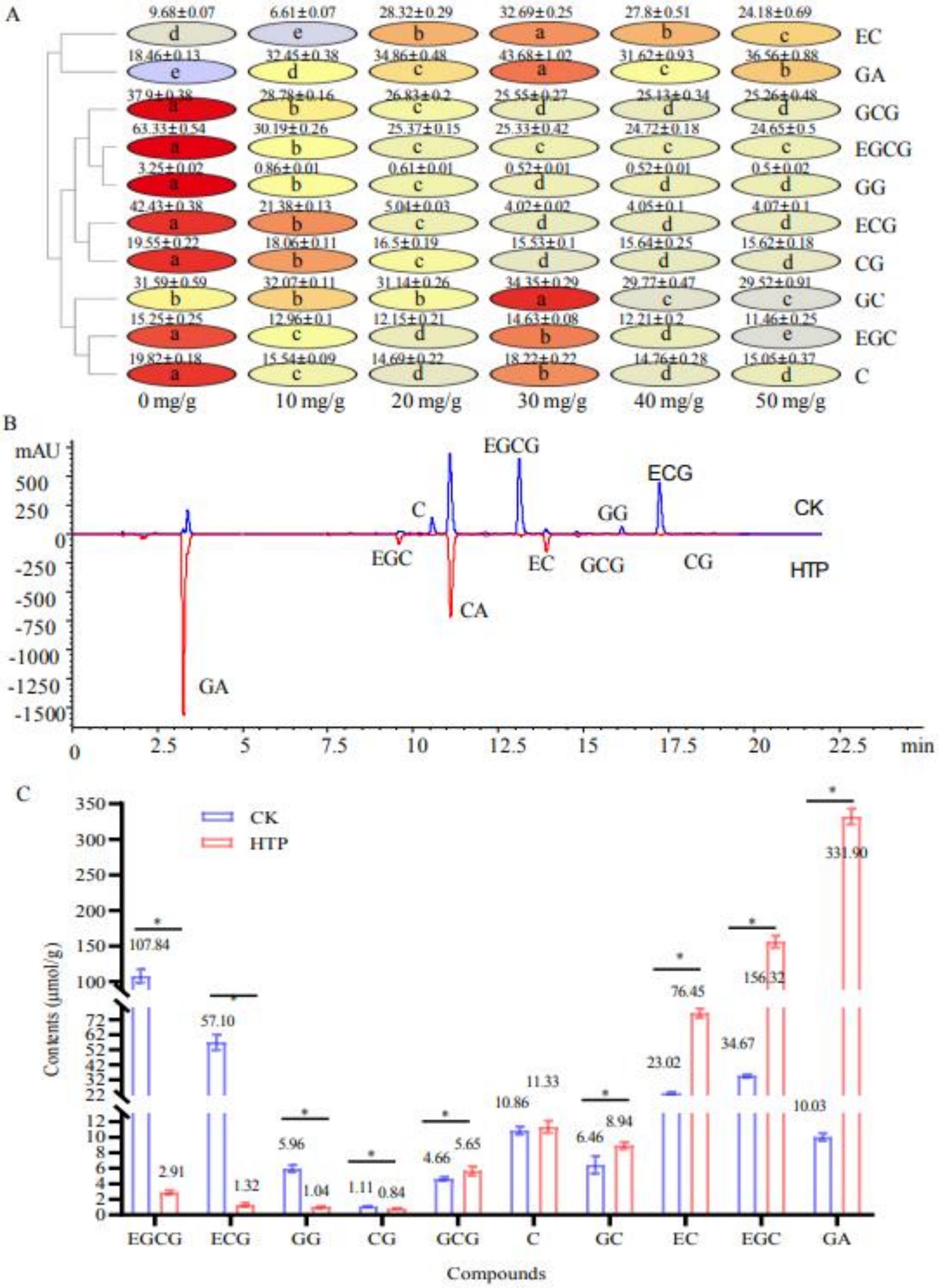

Fig. S4. High-performance liquid chromatography (HPLC) analysis of the 610 metabolism of tea gallates by tannase. Concentrations of gallates, catechins, and gallic 611 acid (GA) in fermented tea leaves with tannase (A). HPLC chromatograms for determination of compounds in tea powders (B). Comparison of concentrations of gallates, catechins, and GA in tannase-hydrolyzed tea powders and controls (C). Different lowercase letters in (A) and * in (C) indicate a significant difference between the contents (Liu, Xie, Ma, Li, Li, Chen, ... & Zhao, 2020).

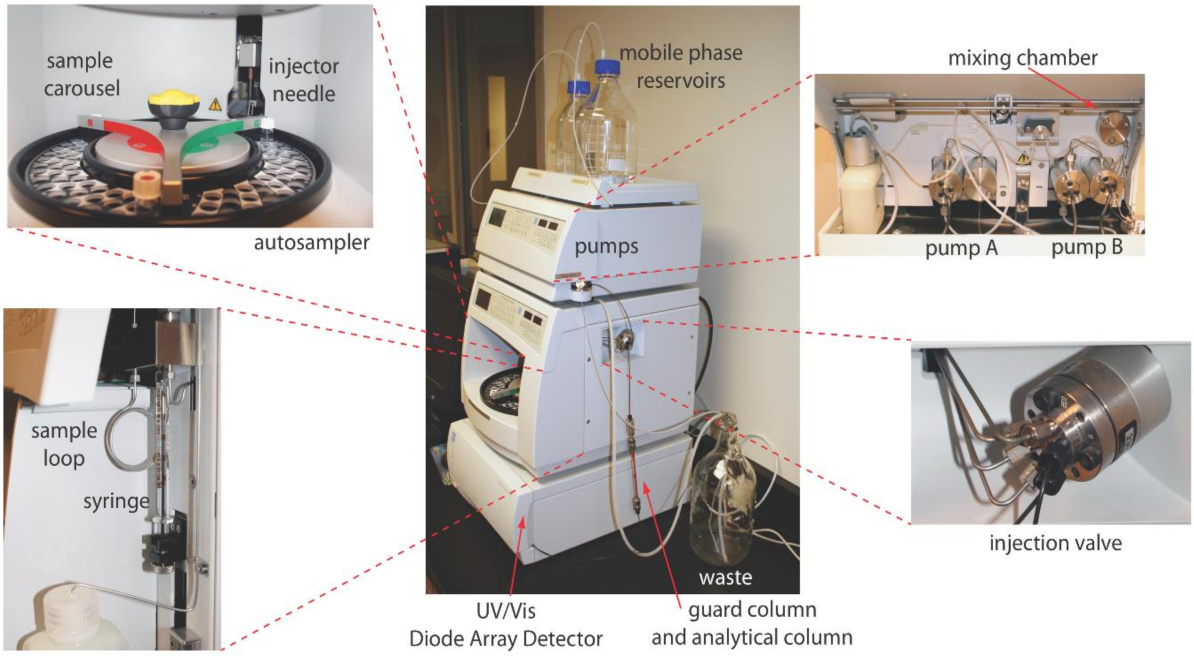

Fig. S5. Example of a typical high-performance liquid chromatograph (Chem LibreTexts, 2024).

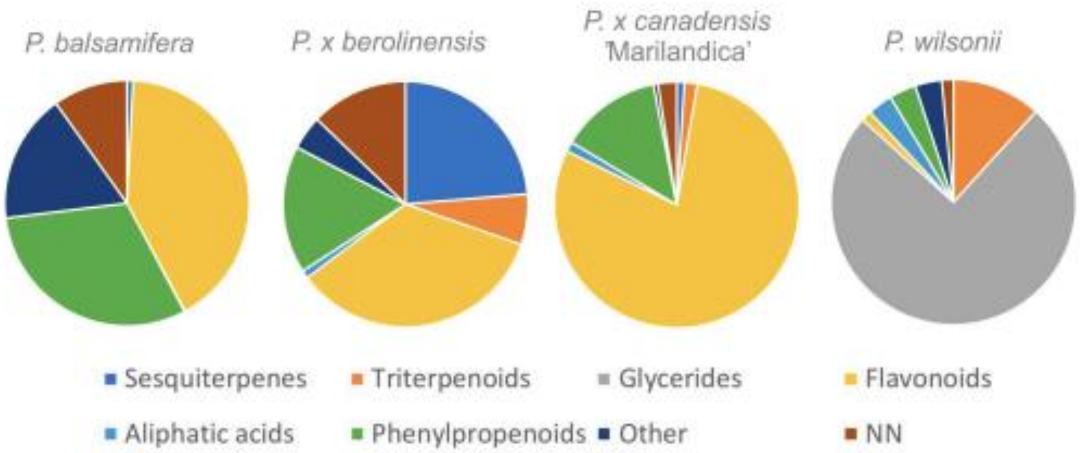

Fig. S6. The content (% of TIC) of the main group of compounds identified by the GC-MS method in analyzed poplar buds (Pobłocka-Olech, Isidorov, & Krauze-Baranowska, 2024).

Supplementary References

[1] Garg R, Maru G. Dietary curcumin enhances benzo(a)pyrene-induced apoptosis resulting in a decrease in BPDE-DNA adducts in mice. J Environ Pathol Toxic ol Oncol 2009, 28: 121-131.

[2] Jamshidi-Kia F, Wibowo J P, Elachouri M, et al. The battle between plants as antioxidants with free radicals in the human body. Journal of Herbmed Pharma cology, 2020, 9(3): 191-199.

[3] Salami S A, Guinguina A, Agboola J O, et al. In vivo and postmortem effects of feed antioxidants in livestock: a review of the implications on authorizatio n of antioxidant feed additives[J]. Animal, 2016, 10(8): 1375-1390.

[4] Liu M, Xie H, Ma Y, et al. High performance liquid chromatography and met abolomics analysis of tannase metabolism of gallic acid and gallates in tea lea ves[J]. Journal of agricultural and food chemistry, 2020, 68(17): 4946-4954.

[5] Chem LibreTexts. (2024). *High-performance liquid chromatography*. LibreTexts.

[6] Olech P L, Isidorov A V, Baranowska K M. Characterization of Secondary Metabolites of Leaf Buds from Some Species and Hybrids ofPopulusby Gas Chromatography Coupled with Mass Detection and Two-Dimensional High-Performance Thin-Layer Chromatography Methods with Assessment of Their Antioxidant Activity. International Journal of Molecular Sciences, 2024, 25(7): 3971.
